# Understanding off-target growth defects introduced to influenza A virus by synonymous recoding

**DOI:** 10.1261/rna.080675.125

**Published:** 2025-11

**Authors:** Colin P. Sharp, Beth H. Thompson, Ananya Ferdous Hoque, Ola Diebold, Blanka Tesla, Dominic Kurian, Peter Simmonds, Paul Digard, Eleanor Gaunt

**Affiliations:** 1 The Roslin Institute, The University of Edinburgh, Easter Bush Campus, Midlothian EH25 9RG, United Kingdom; 2 Cambridge Institute for Therapeutic Immunology and Infectious Disease, Jeffrey Cheah Biomedical Centre, Cambridge Biomedical Campus, Cambridge CB2 0AW, United Kingdom; 3 Wellcome Centre for Human Genetics, Nuffield Department of Medicine, Oxford University, Oxford OX3 7BN, United Kingdom; 4 Institute of Biomedicine, University of Turku, FI-20520 Turku, Finland

**Keywords:** influenza virus, RNA polymerase, synonymous recoding, frameshift

## Abstract

CpG dinucleotides are underrepresented in the genomes of most RNA viruses. Synonymously increasing CpG content of a range of RNA virus genomes reliably causes replication defects due to the recognition of CpG motifs in RNA by cellular zinc-finger antiviral protein (ZAP). Prior to the discovery of ZAP as a CpG sensor, we described an engineered influenza A virus (IAV) enriched for CpGs in segment 5 that displays the expected replication defects. However, we report here that this CpG-high (“CpGH”) mutant is not attenuated by ZAP. Instead, a pair of compensatory nucleotide changes, resulting in a stretch of eight consecutive adenosines (8A), were found to be responsible. Viral polymerase slippage occurs at this site, resulting in the production of aberrant peptides and type I interferon induction. When the nucleotides in either one of these two positions were restored to wild-type sequence, no viral attenuation was seen, despite the 86 extra CpGs encoded by this virus. Introduction of these two adenosines into wild-type virus (thereby introducing the 8A tract) resulted in viral attenuation, polymerase slippage, aberrant peptide production and type I interferon induction. That a single nucleotide change can offset the growth defects in a virus designed to have a formidable barrier to wild-type reversion highlights the importance of understanding the processes underlying viral attenuation. Poly(A) tracts are a correlate for the emergence of polybasic cleavage sites in avian IAV hemagglutinins to produce highly pathogenic strains. These results thereby uncover possible insights into the intermediary events of this important evolutionary process.

## INTRODUCTION

To proliferate, viruses must efficiently hijack the host translation machinery to make their own proteins, while subverting cellular antiviral sensors. The evolutionary pressures that these two requirements impart conditions viral genes, manifesting as compositional biases in the genome that become ingrained over evolutionary time (Greenbaum et al. 2008). For example, vertebrate genomes suppress CpG, and aberrant CpG patterns in viral transcripts may alert the infected cell to the presence of virus. In human cells, this is accomplished through sensing of CpGs in viral RNAs (vRNAs ) by zinc-finger antiviral protein (ZAP) (Takata et al. 2017). Viruses with negative sense −ssRNA genomes are among those viruses that strongly suppress CpG, thereby mimicking the low genomic CpG content of their vertebrate hosts and evading ZAP-mediated detection (Bird 1980; Cooper and Krawczak 1989; Simmonds et al. 2013).

Adding CpGs into viral genomes is a now well-characterized attenuation mechanism that has been proposed by ourselves and others to have potential application in live, attenuated vaccine design (Atkinson et al. 2014; Tulloch et al. 2014; Simmonds et al. 2015; Gaunt et al. 2016; Antzin-Anduetza et al. 2017; Fros et al. 2017; Takata et al. 2017; Digard et al. 2020; Gaunt and Digard 2021). Using influenza A virus (IAV) as a tractable model system in which to test this, we recently reported that increasing the CpG content of segment 1 of the IAV genome resulted in a ZAP-mediated attenuation that caused CpG-high (CpGH) transcript turnover, but not type I interferon induction (Sharp et al. 2023), despite ZAP's identity as an interferon-stimulated gene.

Synonymous recoding of viral genomes, including CpG enrichment, is founded on the principle that attenuation is mediated through multiple nucleotide changes, reducing the potential for reversion to wild-type (WT) sequence. However, this does not guarantee that the recoded virus will not revert to WT virus fitness. For example, if an RNA structure in a viral genome is required for optimal virus replication, adding CpG dinucleotides would distort that structure, and the virus would be attenuated. It would appear as though the recoded virus was attenuated due to the added CpGs, but in reality, the attenuation was mediated by one or two nucleotide changes that distort an RNA structure out of the hundreds made. As an historical example of this, a single nucleotide change in the Sabin type 3 live attenuated poliovirus vaccine 5′UTR restored an internal ribosome entry site, improving virus fitness sufficiently to lead to rare, sporadic cases of acute poliomyelitis in some vaccine recipients (Svitkin et al. 1990). This highlights the importance of confirming the mechanism when using nucleotide substitutions to impart viral attenuation.

Prior to the discovery of ZAP as a CpG sensor, we reported that adding CpGs to segment 5 of the A/Puerto Rico/8/1934 (PR8) strain of IAV caused viral attenuation (Gaunt et al. 2016). Here, we have tested the sensitivity of this virus to ZAP. Unexpectedly, virus attenuation was not abrogated by ZAP knockout. We find that instead, compensatory mutations made to maintain individual nucleotide frequencies resulted in the introduction of an 8-adenosine (8A) stretch, which was responsible for the attenuated phenotype due to IAV polymerase slippage leading to aberrant protein production, and an enhanced type I interferon response. Attenuation was alleviated by disruption of the 8A motif, and introduction of these two point mutations into the WT virus phenocopied the original mutant. This study is an important warning, pertinent to the growing interest in using large-scale synonymous virus deoptimization in vaccinology.

## RESULTS

### CpG enrichment in segment 5 of the IAV genome results in ZAP-independent attenuation

**FIGURE 1.**
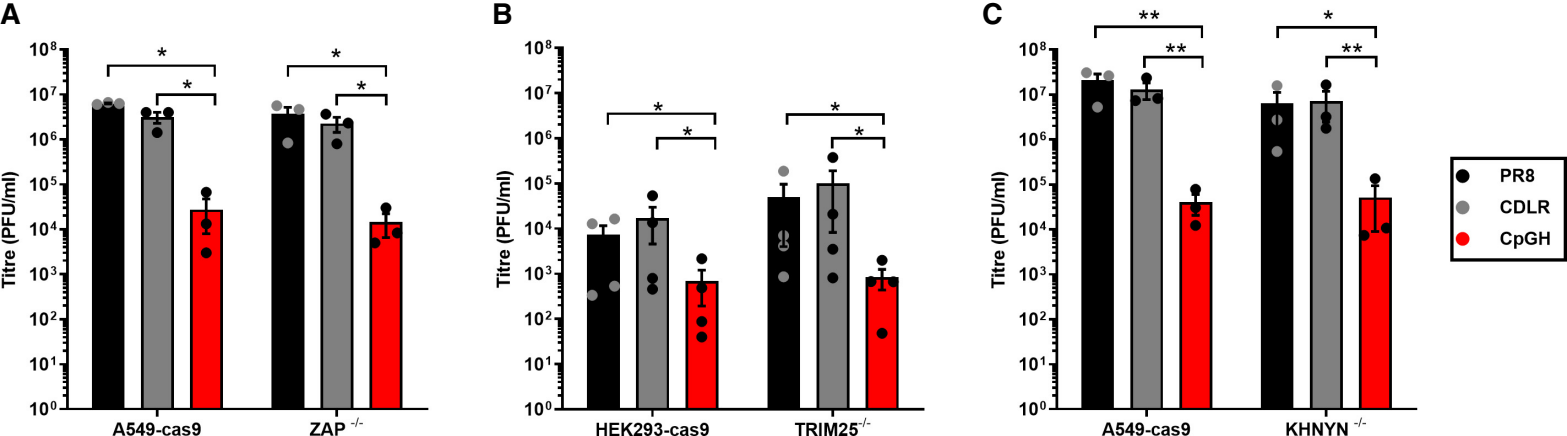
CpG enrichment in segment 5 of the IAV genome results in ZAP-independent attenuation. WT PR8, CDLR control, and CpGH viruses were used to infect permissive cells and counterpart ZAP-pathway knockout cells at MOI 0.01 for 48 h, and infectious virus production was measured. (*A*) A549-cas9 or paired ZAP−/− cell infections. (*B*) HEK293-cas9 or paired TRIM25−/− cell infections. (*C*) A549-cas9 or paired KHNYN−/− cell infections.

Prior to the discovery of ZAP as the cellular sensor of CpG dinucleotides (Takata et al. 2017), we had reported the use of CpG enrichment in segment 5 of the PR8 strain of IAV as a successful attenuation approach (Gaunt et al. 2016). We therefore tested whether our CpGH IAV was attenuated in ZAP-deficient systems. The virus panel comprised WT PR8, a permuted control with reordered codons but equal dinucleotide composition (CDLR), and CpGH virus, which had 86 CpGs added and further compensatory mutations to retain nucleotide frequencies (Table 1). As we previously reported (Gaunt et al. 2016), growth of the CpGH virus was significantly attenuated in A549-cas9 cells, yielding end point titers ∼2log_10_ lower than WT and control viruses. However, in paired A549 ZAP−/− cells, the fitness defect of the CpGH virus was retained (Fig. 1A). Similarly, knockout of cofactors in the CpG sensing pathway could not rescue the defective replication of this CpGH virus, either when TRIM25 was knocked out of HEK293 cells (Fig. 1B), or KHNYN was knocked out of A549 cells (Fig. 1C). The replication defect of the CpGH PR8 virus was therefore not due to ZAP-mediated sensing.

**TABLE 1.** Properties of CpG modified PR8 IAVs recoded in segment 5

Mutation	Recoded region	No. CpGs	ΔCpG	No. UpAs	Δ UpA
Wild-type segment 5	—	43	—	52	—
CDLR	159–1404	43	—	52	—
CpGH	162–1413	129	+86	54	+2
CpGH/A.PR8	628–1413	100	+57	53	+1
CpGH/B.PR8	151–624; 986–1413	110	+67	48	−4
CpGH/C.PR8	151–986	91	+48	58	+6
CpGH/D.PR8	151–297; 783–1413	98	+55	49	−3
CpGH/E.PR8	151–780; 1283–1413	94	+51	53	+1

### CpG enrichment in segment 5 of the IAV genome does not impair viral packaging

**FIGURE 2.**
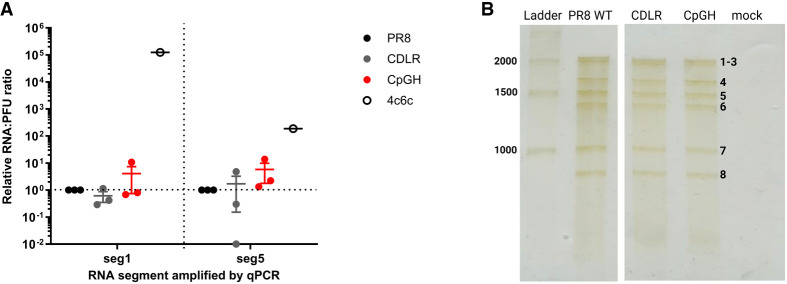
CpG enrichment in segment 5 of the IAV genome does not impair viral packaging. (*A*) RNA was extracted from 10^7^ PFU virus stocks for WT PR8, CDLR control, and CpGH viruses, and relative copy numbers of segment 1 and segment 5 RNA were quantified by qPCR. A “4c6c” packaging mutant was used as a negative control. (*B*) 10^10^ PFU of egg-derived virus panel virions were purified, then genomic RNA was extracted, and individual segments were separated using urea-PAGE and visualized by silver staining. Numbers indicate location of each genome segment. Mock is RNA from the allantoic fluid of uninfected eggs, prepared using the same methods.

We considered whether the CpGH virus may be attenuated due to a genome packaging defect. To test this, RNA was extracted from semipurified virions, and RT-qPCR was performed to determine the relative amounts of segment 1 (encoding PB2) and segment 5 (encoding NP) RNA in virions. No differences could be found across the panel, in contrast with a known packaging mutant “4c6c” (Wise et al. 2019), for which more copies of both segment 1 and segment 5 RNA were needed to produce an infectious virus (Fig. 2A). As a second check of packaging, genomic RNA from semipurified virions was inspected visually using urea-PAGE electrophoresis. No differences were observed in band density for any segments of any of the viruses in the panel (Fig. 2B), confirming that CpG enrichment in segment 5 did not impair IAV genome packaging.

### CpG enrichment in segment 5 of the IAV genome reduces NP transcript and protein abundance in cells

**FIGURE 3.**
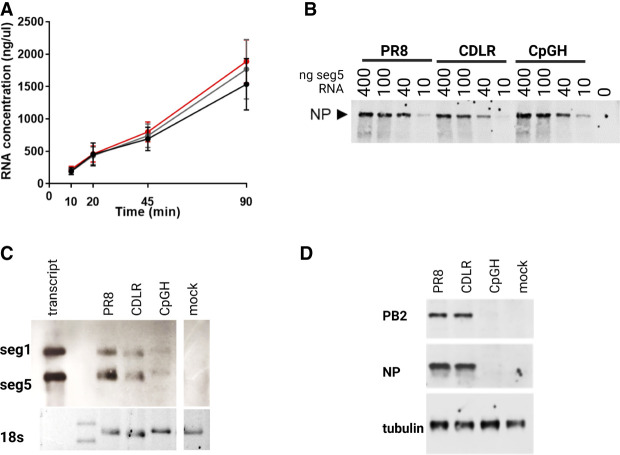
CpG enrichment in segment 5 of the IAV genome reduces NP transcript and protein abundance in cells. (*A*) To assay transcription efficiency, in vitro transcription assays using T7 RNA polymerase were performed using the segment 5 plasmids, with RNA quantified by Qubit at 10, 20, 45, and 90 min. (*B*) To assay combined transcription and translation in a cell-free system, limiting dilutions of segment 5 plasmids were used in rabbit reticulocyte lysate coupled transcription and translation assays, and NP protein was detected by western blotting using an NP-specific antibody. (*C*, *D*) A549 cells were infected at MOI 10 for 10 h, then RNA abundance was examined by northern blotting (*C*) and protein abundance by western blotting for the indicated species (*D*).

We considered whether CpG enrichment had caused a defect in transcription and/or translation. Firstly, we tested transcript yield from the CpGH construct in an in vitro transcription assay (with RNA produced from DNA amplicons under a T7 RNA polymerase promoter); no differences were observed across the panel (Fig. 3A). Similarly, no differences in protein levels were detected in in vitro translation assays (Fig. 3B). Next, we examined transcript and protein production during virus infection. A549 cells were infected at multiplicity of infection (MOI) 10, and after a full replication cycle, total RNAs were harvested and positive sense vRNAs were assayed using northern blotting (Fig. 3C). Transcript levels for the CpGH virus were down for both segment 5 and segment 1. This correlated with reduced virus protein levels (Fig. 3D). The difference in RNA and protein abundance in the cell-free versus the infection assays indicated that either cellular factors were reducing segment 5 yield, or that the IAV polymerase could not process the segment 5 RNA in the same way as the T7 RNA polymerase.

### Tiled reversion of the CpGH sequence to wild-type sequence identifies a short region that when restored to wild-type sequence, reconstitutes wild-type virus fitness

**FIGURE 4.**
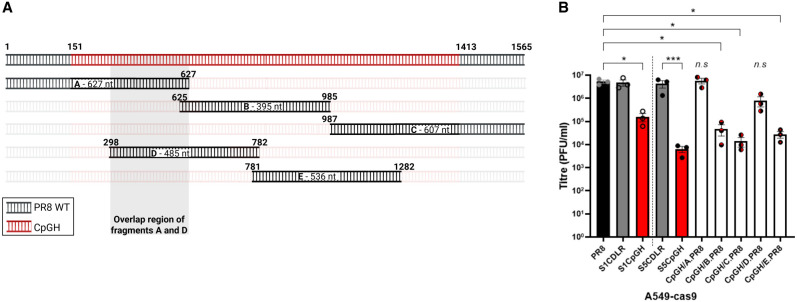
Tiled reversion of the segment 5 CpGH sequence to WT identifies a short region that is common to viruses with WT virus fitness. (*A*) To identify the recoded region of the CpGH virus contributing the attenuated phenotype, overlapping fragments A–E were reverted to WT PR8 sequence as indicated. (*B*) Fragmented reversion viruses (e.g., CpGH virus with fragment A reverted to WT PR8 sequence, denoted as CpGH/A.PR8) were used to infect A549-cas9 cells at MOI 0.01 for 48 h, and virus production was measured.

To gain insights into the mechanism by which the CpGH virus was attenuated, we sought to identify the recoded region imparting viral attenuation. Segment 5 CpGH was split into five fragments, A–E, with A–C forming three sequential fragments covering the full length of the transcript, and D and E mapping across the overlap regions. A panel of viruses was made where each fragment was reverted to WT PR8 sequence (CpGH/A.PR8, etc.) (Fig. 4A; Table 1). MOI 0.01 infections were then performed in A549 cells to assess virus fitness. CpGH viruses with fragments B, C, or E reverted to PR8 WT sequence maintained a replication defect similar to the CpGH virus, suggesting that mutations applied in these regions were not essential for attenuation. However, CpGH/A.PR8 recovered replication fitness to levels similar to the WT PR8 virus (Fig. 4B), indicating that the defect in the CpGH virus was imparted by mutation(s) applied in this region. Recovery of fitness for the CpGH/D.PR8 virus was also evident. Notably, fragments A and fragments D overlap between nucleotides 298 and 626 (Fig. 4A), indicating that mutations in the overlap region may be responsible for the replication defect of CpGH virus.

### Serial passage of segment 5 CpGH IAV identifies reversion mutations that fall within an 8 nucleotide stretch of adenosines

**FIGURE 5.**
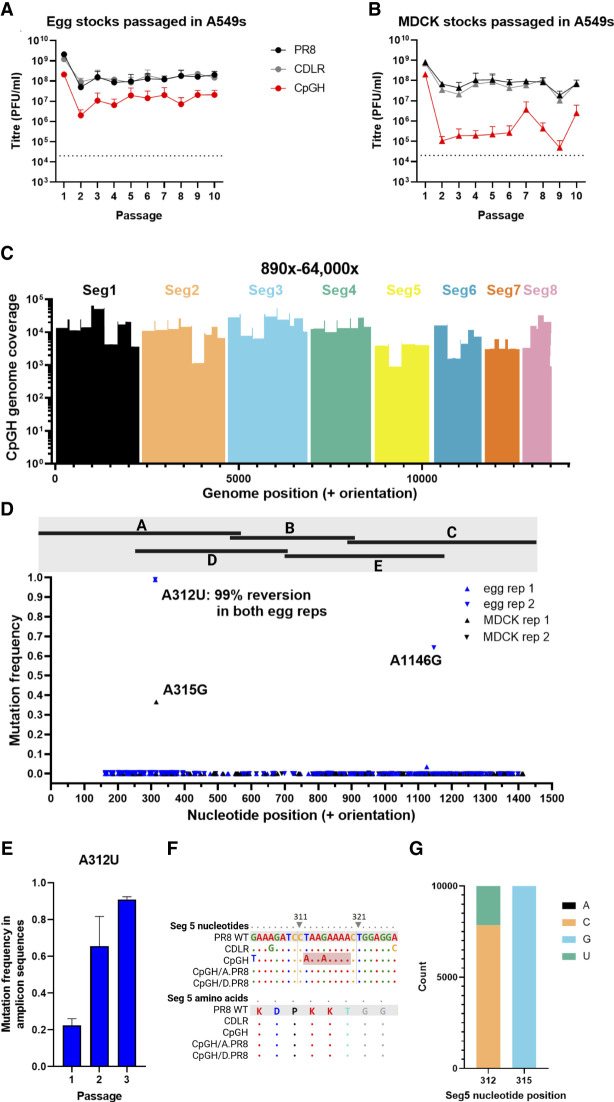
Serial passage of segment 5 CpGH IAV identifies reversion mutations that fall within an 8 nucleotide stretch of adenosines. Virus panel generated from either egg (*A*) or MDCK (*B*) rescue was serially passaged 10 times at MOI 0.01 in A549 cells, with virus titered after each passage, for four biological repeats (two with starting inoculum of egg rescue and two of MDCK). At passage 10, virus was deep sequenced. (*C*) Read depth from deep sequencing of CpGH virus. (*D*) Mutations occurring exclusively in the CpGH virus were plotted. None of these mutations occurred at CpG sites. Their position relative to fragments A–E (Fig. 4) are indicated by the black bars at the head of the panel. (*E*) Serially passaged viruses derived from egg rescue were sequenced after 1, 2, and 3 passages; for the CpGH virus, mutation frequency at position 312 is shown. (*F*) Reversion mutations at positions 312 and 315 corresponded with an 8-adenosine tract introduced exclusively into the CpGH virus. (*G*) Variability of segment 5 nucleotide positions in nature (10,000 human sequence isolates analyzed over 5 years between 2018 and 2022).

To try and identify point mutations responsible for the ZAP-independent attenuation, we performed serial passage experiments. Viruses were passaged 10 times at MOI 0.01 on A549 cells, and then deep sequencing was performed. Titers did not recover for CpGH virus stocks grown in either eggs (Fig. 5A) or MDCK cells (Fig. 5B). Deep sequencing yielded coverage across the genome between 890× and 64,000× (Fig. 5C). While no nucleotide reversions were seen at sites where CpG dinucleotides had been introduced, a single nucleotide reversion occurred in 98.8% of sequencing reads from both egg virus rescues, at nucleotide position 312. An adjacent nucleotide reversion was also seen in one of the MDCK stock rescues, with ∼40% of reads yielding a reversion at position 315 (Fig. 5D). Both of these reversions occurred within the overlap region of fragments A and D between nucleotides 298 and 627 (indicated on Fig. 4A). To determine when during passage the A312U mutation arose, PCR amplification across this region was performed on RNA extracted from the P1 viral stock, and after a further one or two passages (P2 and P3), the amplicons were deep sequenced. This sequencing showed that the mutation at nucleotide position 312 was present in the P1 viral stock (∼20% of reads), becoming the dominant variant in the next passage and increasing to represent ∼90% of reads by P3 (Fig. 5E), suggestive of strong positive selection in favor of this mutation. Visual inspection of nucleotide alignments revealed that the CpGH virus incorporated an 8-adenosine (8A) stretch that included nucleotide positions 312 and 315, because of mutations added to balance compositional changes involved in adding CpGs (Fig. 5F). In over 10,000 human IAV H1N1 isolate sequences downloaded from GenBank and analyzed, no adenosines were seen at positions 312 or 315, suggesting the 8A stretch rarely if ever occurs in nature (Fig. 5G). While sequence reversion at the 8A site was observed, the CpGH virus still displayed a replication defect after reversion at this site (Fig. 5A,B), indicating multilayered attenuation.

### Single nucleotide reversions at positions 312 and 315 of the CpGH virus restored WT fitness

**FIGURE 6.**
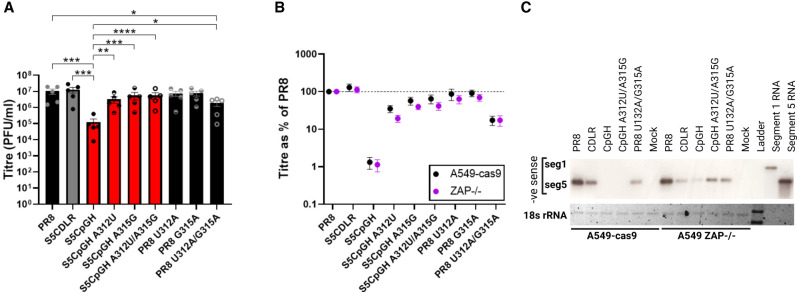
Single nucleotide reversions at positions 312 and 315 of the CpGH virus restored WT fitness. The CpGH virus (S5CpGH) incorporated an 8A tract in the coding sequence. This was removed by reversion to WT sequence at these nucleotide positions through mutations A312U and/or A315G. Conversely, the poly(A) stretch was introduced into WT PR8 via U312A and/or G315A mutations. (*A*) Titer of virus panel grown at MOI 0.01 for 48 h in A549 cells. (*B*) To determine whether ZAP sensing was apparent for any mutants, the virus panel was grown in A549-cas9 or ZAP−/− cells, and titers were normalized to WT PR8 titers. (*C*) To determine whether input RNA from incoming virions was degraded, A549-cas9 cells were infected with the virus panel at MOI 10 in the presence of cycloheximide; RNA was harvested at 24 h postinfection, electrophoresed, and northern blotted using probes for IAV segments 1 and 5 (negative orientation). Ribosomal RNA (rRNA) served as a loading control.

The tiled reversion data (Fig. 4) and serial passage data (Fig. 5) taken together suggest that the ZAP-independent attenuation of CpGH virus was due to the nucleotide changes at positions 312 and/or 315. Therefore, we made CpGH viruses with these nucleotides reverted singly and in combination, and assessed their replicative fitness in A549 cells. As previously, the CpGH virus was defective in comparison with WT and CDLR control viruses (Fig. 6A). However, when the 8A tract in CpGH was disrupted by reverting A to U at position 312 (S5CpGH A312U), or the A at position 315 to G (S5 CpGH A315G), the replication defect was abrogated. The same phenotype was observed for the double mutant (S5CpGH A312U/A315G). When these single nucleotide changes were built into otherwise WT PR8, neither PR8 U312A or PR8 G315A were attenuated, but when both mutations were introduced together (PR8 U312A/G315A) to reconstitute the 8A tract, the virus was significantly attenuated compared to WT PR8. Notably, the CpGH virus, which incorporated both 8A and CpG enrichment, was more attenuated than PR8 U312A/G315A, indicating the presence of two independent and complementary attenuation mechanisms.

To determine whether ZAP sensing was evident for any of the CpGH viruses, the same panel was grown in A549 ZAP−/− cells. Titers were normalized to PR8 for WT versus ZAP−/− cell lines for direct comparison. However, no improvement in ZAP−/− cells was observed for any of the viruses in the panel (Fig. 6B). Therefore, we sought to determine whether the transcript abundance was diminished due to the added CpG dinucleotides, the 8A tract, or a combination of both. Depletion of segment 5 transcript can reasonably be expected to impact downstream vRNP production and consequently overall virus gene expression. Thus, to ensure equivalent numbers of vRNPs across the RNA abundance assay, A549 cells were infected at MOI 10 for 10 h in the presence of cycloheximide, which blocks translational elongation/protein synthesis thereby preventing nascent vRNP formation, in both A549-cas9 and paired ZAP−/− cells. Northern blotting was performed to assay for primary messenger RNA (mRNA) transcription from incoming vRNPs (complementary RNA [cRNA] will not be produced in the presence of cycloheximide).

Throughout, segment 1 mRNA abundance was below the detection limit of the assay. However, variability in segment 5 abundance was observed (Fig. 6C). In A549-cas9 cells, segment 5 transcript was readily detected for both PR8 WT and CDLR control viruses. CpGH transcript was not detected, indicating its degradation. When the 8A tract was removed but the CpGH profile was maintained (CpGH A312U/A315G), no RNA was detected, suggesting that it was specifically the CpG motifs that resulted in transcript degradation. When the 8A tract was added into the WT PR8 segment 5 (PR8 U312U/G315A), RNA was detected, but it was of lower abundance than for WT PR8, suggesting that the 8A tract also resulted in increased transcript turnover. In ZAP−/− cells, the detection of segment 5 transcripts for both CpGH and CpGH A312U/A315G was improved, consistent with ZAP-dependent degradation of CpG-enriched transcripts. This was in keeping with our previous observations of ZAP-mediated CpGH transcript degradation (Sharp et al. 2023), but in segment 5, this alone was evidently insufficient to impair viral replication (Fig. 6B).

### Introduction of the 8A tract into IAV segment 5 resulted in viral polymerase slippage, aberrant protein production, and triggering of type I IFN

**FIGURE 7.**
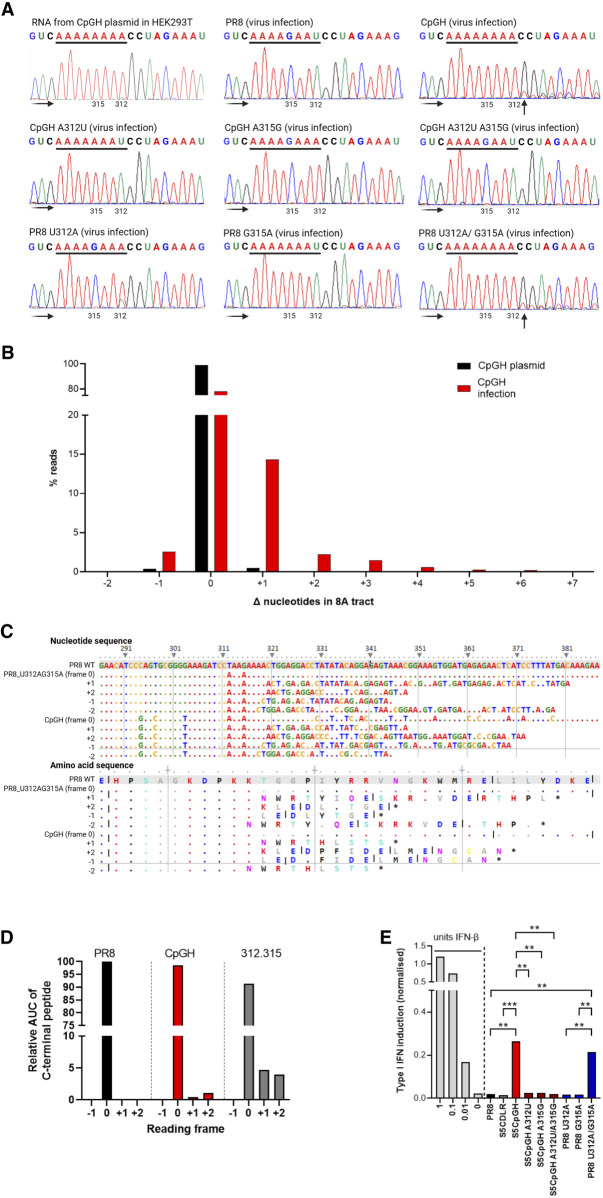
Introduction of the 8A tract into IAV segment 5 resulted in viral polymerase slippage, aberrant protein production, and triggered type I IFN. (*A*) WT PR8, CpGH, and cognate viruses with switched nucleotides at positions 312 and 315 were used to infect A549 cells at MOI 3 for 8 h, after which RNA was extracted, amplified, and sequenced. Chromatogram traces were examined for evidence of multiple RNA species generated downstream from the 8A sequence of CpGH virus, encoded at nucleotide positions 312–319. Faded black arrows indicate direction of sequencing read. Solid black arrows indicate sites of polymerase slippage. (*Top left*) CpGH plasmid was transfected into HEK293T cells, and +RNA was produced from a pol.II promoter. (*Top middle*) WT PR8 virus infection. (*Top right*) CpGH virus infection. (*Middle left*) CpGH A312U virus (7A tract) infection. (*Middle middle*) CpGH A315G virus (4A tract) infection. (*Middle right*) CpGH A312U A 315G (4A tract) virus infection. (*Bottom left*) PR8 U312A virus (4A tract) infection. (*Bottom middle*) PR8 virus G315A (7A tract) infection. (*Bottom right*) PR8 U312A G315A virus (8A tract) infection. (*B*) CpGH plasmids and infections were deep sequenced, and percentage of sequence reads with changes in the length of the 8A tract were calculated. (*C*) *Top* panel—Nucleotide alignment of PR8, PR8 G312A U315A, and CpGH sequence surrounding the poly(A) site, with alignments to show the nucleotide sequence resulting from +1, +2, −1, and −2 polymerase slippage events. The *bottom* panel shows the resulting peptide species arising from these transcripts. (*D*) To examine for the presence of frameshifted peptide production, A549 cells were infected at MOI 3 for 8 h with either WT PR8, CpGH, or PR8 A312U A315G viruses, and NP peptide production was assessed using mass spectrometry to examine for a shift into alternative reading frames. Due to differences in m/z ratios for the peptides unique to +1 and +2 frameshifted translations, relative abundance cannot be compared across peptide species. The −1 frameshift followed by gluC digestion resulted in predicted peptides that were not of sufficient length for detection by mass spectrometry. No peptides predicted to have arisen from the −2 frameshift were detected. (*E*) Type I interferon competent A549 cells were infected with virus panel for 10 h at MOI 10, after which time supernatant was harvested, UV treated to inactivate infectious virus and assayed for IFN content using HEK Blue cells. HEK Blue cells were also treated with IFN standard (light gray bars). Means of three biological repeats are shown.

To generate the poly(A) tract at the 3′ end of viral mRNAs, the IAV polymerase is known to slip on a poly(U) stretch (Poon et al. 1999), which occurs at the 3′ termini proximal to the panhandle structure of genome segments. We considered the possibility that in the CpGH construct, the viral polymerase was slipping at 8A in the middle of the segment, resulting in aberrant transcript production. To test this, we examined chromatograms of viral transcript sequences when synthesized by different RNA polymerases. Firstly, the CpGH plasmid was transfected into HEK293T cells, and extracted RNA was DNase treated, amplified by RT-PCR across the 312/315 region and sequenced to assess for aberrant transcript production. The sequencing chromatogram indicated no evidence of secondary nucleotide peaks around the 8A tract, indicating that RNA polymerase II did not slip on the 8A sequence (Fig. 7A, top-left panel). Similarly, when A549 cells were infected with WT PR8 virus, the viral +RNA yielded a clean chromatogram for RNA synthesized by the viral polymerase (Fig. 7A, top-middle panel). However, when the same cells were infected with CpGH virus, sequencing plus strand vRNA, there was a mixed transcript pool represented in the chromatogram downstream from the 8A tract (Fig. 7A, top-right panel). This suggested that the mixed transcript population most likely arose through vRNA polymerase slippage. When the A base at nucleotide position 312 was reverted to a U (as in the WT PR8 sequence), evidence of secondary transcript production was absent, indicating that seven repeated adenosines were insufficient to generate a detectable slippage event (Fig 7A, middle-left panel). Similarly, evidence of slippage was absent from CpGH A315G (containing nucleotide sequence AAAGAAA; Fig. 7A, middle panel) and from CpGH A312U A315G (Fig. 7A, middle-right panel). Finally, the 8A tract was introduced step-wise into the WT PR8 virus; when either U312A or G315A single substitutions were made, no slippage was evident (Fig. 7A, bottom-left and bottom-middle panels). However, when both changes were made together, reconstituting the 8A sequence in PR8 +RNA, the mixed transcript population was again evident in the chromatogram (Fig. 7A, bottom-right panel). Together, these data indicate that a sequence of eight adenosines gives rise to a mixed transcript population, and that seven consecutive adenosines are insufficient to yield this effect.

Deep sequencing of transcripts derived directly from CpGH plasmid transfection versus from virus infection confirmed the increased proportion of sequences containing evidence of viral polymerase slippage on the 8A site in the context of infection (Fig. 7B). To assess the consequences of IAV polymerase slippage at 8A on protein production, cell lysates from WT PR8, CpGH, and PR8 U312A G315A infections were analyzed using mass spectrometry to determine whether aberrant proteins were produced. Infected cells were treated with MG132, a protease inhibitor, to prevent turnover of misfolded proteins. Due to the 8A stretch introducing multiple lysines, this meant that the standard approach of tryptic cleavage would not allow us to distinguish between +1 and −2, or +2 and −1 frameshift events, the gluC protease that cleaves downstream from glutamic acid was used instead. Sequencing data (Fig. 7B) indicated that frameshifts were most likely to occur in a +1, +2, or −1 orientation, and so the predicted protein translations resulting from such shifts (Fig. 7C) were searched for. For WT PR8, only peptides in the canonical reading frame were identified (Fig. 7D). However, for both CpGH virus and PR8 U312A G315A, canonical, +1 and +2 frame peptides were identified. No −1 frame peptides were identified in any samples. Due to differences in m/z ratios for the peptides unique to +1 and +2 frameshifted translations, relative abundance should not be compared across peptide species, and so where relative abundances of C-terminal peptides are compared, this should be taken as approximate (Carrillo et al. 2010). Nevertheless, the mass spectrometry confirmed that IAV polymerase slippage resulted in production of aberrant NP polypeptides.

We considered the possibility that aberrant viral transcriptional and translational events may result in the induction of type I interferon, which was investigated using HEK Blue assays. As expected due to the potent type I interferon blocking activity of NS1 (García-Sastre et al. 1998), WT PR8 and CDLR control virus infections in A549 cells did not induce interferon above baseline (Fig. 7E). Conversely, 8A-containing CpGH virus significantly induced interferon, and this induction was abrogated in the CpGH A312U, A315G and double mutants. Similarly, the PR8 A312U A315G double mutant (8A-encoding), but not the single mutants, also induced interferon. Together, these data indicate that the 8A tract resulted in viral polymerase slippage, aberrant protein production, and triggering of type I IFN.

### IAV polymerase slippage preferentially occurs on poly(A) over poly(U)

**FIGURE 8.**
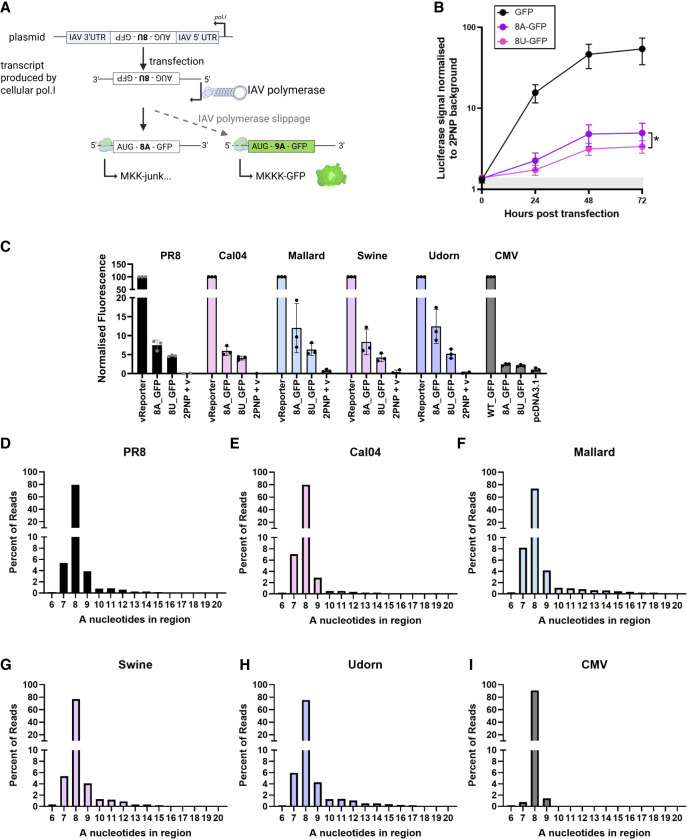
IAV polymerase slippage preferentially occurs on 8U tract over 8A, offering a putative mechanism for the generation of polybasic cleavage sites. (*A*) Two plasmid constructs were designed to encode the EGFP ORF downstream from an 8-adenosine or 8-uracil tract, flanked by PR8 NS1 segment UTRs that are recognized by the viral polymerase and under an RNA pol.I promoter. These constructs were used in a minigenome assay (together with plasmids that together reconstitute viral polymerase) to determine whether polymerase slippage could be evidenced through reporter gene detection. Due to the incorporation of the 8A/8U tract upstream of the GFP ORF, the ORF is out of frame. Polymerase slippage on the 8A/8U resulting in a single nucleotide insertion will put the GFP ORF in frame and yield GFP signal. (*B*) GFP signal was read for in-frame GFP, GFP downstream from an 8A oligonucleotide, and GFP downstream from an 8U oligonucleotide, under IAV promoter and polymerase expression (annotations refer to sequence in positive polarity). Fluorescence was read every 24 h and normalized to reporter transfected with incomplete IAV polymerase components (no PA). (*C*) Relative fluorescence of GFPs encoded downstream from 8A and 8U sequences compared with in-frame GFPs for PR8, Cal04, Mallard, Swine, and Udorn polymerases, and CMV-promoter driven host polymerases. (*D*–*I*) To determine whether polymerase slippage occurred for other IAV polymerases, the IAV PR8, Cal04, Mallard, Swine, Udorn, and human CMV polymerases were used to amplify the transcripts containing 8A upstream of the GFP sequence, and amplicon sequencing was performed to determine the extent of polymerase slippage on the 8A sequence.

During IAV replication, the negative sense genome is copied into positive sense to make mRNA for protein production, and cRNA to serve as a template for vRNA production to synthesize new virions. As the polymerase can synthesize transcripts of both polarities, we considered whether the polymerase may be slipping on the 8A tract of the positive-sense transcript, and/or the 8U tract of the negative-sense transcript. To differentiate between the two, a GFP reporter construct with flanking IAV UTRs was separated from its AUG start codon by either an 8A or an 8U tract. The GFP reporter plasmid was designed such that RNA pol.I-mediated transcription would produce negative sense IAV-like transcripts that can be copied into the positive polarity by the IAV polymerase. In this way, polymerase slippage on the upstream 8A/8U resulting in the incorporation of an additional nucleotide would be required to yield GFP signal (Fig. 8A). Upon cotransfection of the GFP plasmid with plasmids expressing the viral polymerase and NP proteins (together required for vRNP formation), GFP signal was read at 24, 48, and 72 h and normalized to background signal (GFP reporter with the polymerase components PB2, PB1, and NP but no PA). The WT in-frame GFP construct yielded increasing signal over the course of the experiment, approaching a 100-fold increase in signal (Fig. 8B). For the transcript encoding 8U in the negative polarity (denoted “8A-GFP” in reference to the positive polarity construct sequence), slippage on 8U as the IAV polymerase copies the transcript into the positive polarity would yield GFP signal. For the transcript encoding 8A in the negative polarity (denoted “8U-GFP” in reference to the positive polarity construct sequence), slippage on 8A as the IAV polymerase copies the transcript into the positive polarity would yield GFP signal. Both 8A-GFP and 8U-GFP constructs yielded GFP signal, indicating that in both test systems, a frameshift event was happening to put the GFP sequence in-frame. The signal for 8A-GFP was higher throughout than for 8U-GFP, indicating a preferential slippage on the poly(U). There are two possible explanations for the GFP signal from the 8U-GFP construct. The simplest is that some slippage occurs on the 8A sequence, but this does not occur to the same efficiency as slippage on 8U. Alternatively, it is possible that nascent positive polarity 8U-GFP synthesized from this template may have subsequently been copied to 8A (positive to negative polarity) with slippage occurring, then as 8U was copied back to 8A-GFP (negative to positive polarity), this resulted in in-frame GFP in the positive sense transcript. Thus, IAV polymerase slippage on 8U is certainly apparent, and may also occur at a lower frequency on 8A.

We asked whether PR8 polymerase slippage was specific to this virus, or whether other IAV polymerases would slip in a similar manner. To test this, we replaced the components of the PR8 polymerase used in the experiments described in Figure 8A and B with those from other IAV strains—Cal04, Mallard, Swine, and Udorn—and performed further polymerase reconstitution assays. Evidence of slippage, demonstrated by GFP fluorescence, at 48 h post-transfection was seen for the other four viral polymerases tested (Fig. 8C). This was at a level comparable to PR8. To further examine the direction and extent of polymerase slippage on the 8A sequence, we performed amplicon sequencing. All IAV strains tested had similar slippage profiles, with ∼80% of reads conforming to the original 8A sequence, and ∼5% of transcripts arising from −1 and +1 slippage events (Fig. 8D–H). A “tailing effect” was universally observed, with more and more adenosines added in a decreasing fashion with up to 12 adenosines added (20 in total). Conversely, for the cellular CMV polymerase, <1% of transcripts had evidence of slippage resulting in addition or removal of one adenosine (Fig. 8I). Thus, the IAV polymerase slips more readily than CMV.

## DISCUSSION

CpG enrichment has gained traction as an attractive model for the development or augmentation of live attenuated vaccines (Gaunt and Digard 2021; Sharp et al. 2023). Here, we argue that understanding the mechanism of attenuation imparted by synonymous recoding is imperative as a safety feature of this technology. When we added 86 CpGs to the IAV genome (Gaunt et al. 2016), the virus was attenuated in a way that was apparently phenotypically characteristic of a virus that was being sensed by ZAP. However, through molecular investigations, we have now determined that the replication defects observed primarily arose through an unrelated mechanism requiring a single nucleotide change for fitness restoration. This is therefore a possible scenario during live attenuated vaccine design.

When we added 126 CpGs to segment 1 of the same PR8 IAV, a ZAP-mediated attenuation was imparted, and ∼80 CpGs were sufficient to mediate attenuation. Here, the addition of 86 CpGs without also adding the 8A tract (S5CpGH A312U/A315G) imparted minimal attenuation despite causing a notable reduction in segment 5 transcript abundance. The bigger impact of adding CpGs into segment 1 could simply be attributable to the different expression levels of the two genes (Phan et al. 2021). NP encoded on segment 5 is produced in high abundance (Michalak et al. 2019), and so it is possible that some degradation of viral transcripts is tolerable. This highlights another previously unreported but important consideration for CpG-based recoding designs, as more robust phenotypes may be evident when targeting process-limiting transcripts (Sharp et al. 2023).

Alternatively, RNA structure around CpG sites may determine transcript fate. Binding of CpG motifs by ZAP requires the surrounding RNA to be single stranded (Luo et al. 2020). TRIM25 is required for ZAP's RNA-degrading activity (Li et al. 2017; Zheng et al. 2017), but while TRIM25 is known to bind RNA, no specific motif or structure has been identified for TRIM25 recruitment (Choudhury et al. 2017, 2022). It is unknown whether there is a minimum threshold of ZAP binding to destine transcripts for degradation. CpG enrichment experiments have so far, by necessity, taken crude approaches of adding CpGs to excessive abundance, whereas it may be possible to strategically add fewer CpGs in regions of single-stranded RNA (and without introducing RNA structure) to deliver the same attenuation phenotype.

The finding that the IAV polymerase was slipping at the intra-segment 8U site was unexpected. Such slippage has only been reported on the poly(U) tract at the 5′ end of negative sense RNA (Poon et al. 1999), to produce a polyadenylation signal on viral mRNAs. Here, we provide evidence of polymerase slippage in the middle of a transcript. While our experiments indicate that slippage is more likely to occur on 8U compared with 8A, we cannot determine whether slippage occurs exclusively on 8U, and it was shown by others that if poly(U) at the 5′ end of negative sense RNA was replaced with poly(A), IAV polymerase would also slip on the poly(A) (Poon et al. 1999). Slippage on poly(U) at the segment terminus requires a tract of six consecutive uridines, in contrast with our finding that 8 nt repeat but not a 7 nt repeat induced slippage that was detectable in our assays. This difference could plausibly arise because the poly(U) stretch in the middle of the segment is not positioned adjacent to the template panhandle structure and thus the steric block from the polymerase remaining bound to the 5′-end of the template (Poon et al. 1999). Notably, slippage here is likely to occur at an efficiency that is different from that on terminal poly(U) sequences. Nevertheless, polymerase slippage in the middle of a segment has significant implications for generation of highly pathogenic influenza variants. A key determinant of highly pathogenic influenza strains is the presence of a polybasic cleavage site in the hemagglutinin (HA), which appear to arise spontaneously from low pathogenic strains which have monobasic cleavage sites. An adenosine trinucleotide encodes the basic amino acid lysine, and so it is straightforward to envisage the scenario whereby multiple rounds of IAV polymerase slippage could lengthen a poly(A) tract in the middle of the HA gene, thereby introducing a polylysine (i.e., polybasic) motif. Indeed, adenosine-rich residues appear to be a precursor for polybasic cleavage site emergence (Nao et al. 2017; Kida et al. 2021). Typically, polybasic cleavage sites comprise a combination of lysine and arginine residues, and it may be evolutionarily advantageous for IAVs to introduce single nucleotide changes to replace lysines with arginines (AAA → AGA) to prevent further polymerase slippage. An alternative possibility is spontaneous re-copying, occurring via release and re-priming by the viral polymerase (Perdue et al. 1997).

We found that polymerase slippage ultimately resulted in interferon induction, but the intermediary events connecting these two processes were unclear. Possibly, aberrant transcript or protein production or altered NP abundance stimulates (or suppresses the blockage of) the interferon pathway. The reason why the CpGH virus is attenuated could be due to aberrant transcript production leading to activation of stress response pathways such as nonsense mediated decay, aberrant peptide production accumulation leading to toxicity, type I interferon induction, another mechanism that we have not explored, or could be multifactorial.

With the advent of mRNA vaccine technology, the future of live attenuated vaccines is increasingly uncertain, including for influenza (Singanayagam et al. 2018; Arevalo et al. 2022). Cold-adapted, live attenuated influenza vaccines are typically inoculated in children using nasal sprays (Maassab and DeBorde 1985; Jang and Seong 2012; Chen et al. 2020; Kassianos et al. 2020), thereby avoiding the use of needles; a feature that is unlikely to be achieved by mRNA vaccines. Furthermore, synonymous recoding may have applications in both live-attenuated and in mRNA vaccine development for influenza in the future (Mueller et al. 2010; Yang et al. 2013; Moratorio et al. 2017); for example, contemporary SARS-CoV-2 mRNA vaccines are codon-optimized (Corbett et al. 2020). Here, we highlight the potential pitfalls of recoding-mediated attenuation, with important implications for vaccine safety.

## MATERIALS AND METHODS

### Reagents

Cells were checked monthly for mycoplasma contamination using Lonza MycoAlert kit (Lonza, #LT07-318). For cloning reactions, Gibson assembly kit (New England Biosciences, #M5510A) was used. Plasmids were prepared using QIAGEN midi-prep kit (QIAGEN, #12123). For site-directed mutagenesis, Agilent QuikChange II site-directed mutagenesis kit (Agilent, #200523) was used. Cellular RNA extractions were performed using QIAGEN RNeasy mini extraction kit (#74134) and from supernatants using QIAamp Viral RNA mini kit (#52904) according to the manufacturer's instructions. RNA as visualized after separation on urea-PAGE gels using Bio-Rad Silver Stain Plus Kit (Bio-Rad, #1610449). PCR reactions were undertaken using Q5 High-Fidelity DNA polymerase kits (New England Biosciences, #E0555L). DNA amplicons were extracted from agarose gels using QIAGEN MinElute Gel Extraction Kit (#28604). DNA yield was quantified using Qubit dsDNA Broad Range Assay kit (Thermo Fisher Scientific, #Q32850). In vitro transcriptions were performed using Thermo Fisher Scientific MEGAscript T7 transcription kit (#AM1334). RNA quantification was performed using Thermo Fisher Scientific Qubit RNA High Sensitivity Assay (#Q32852). In vitro translation assays were performed using Rabbit Reticulocyte Lysate kit with one-fifth volume reactions (Promega, #L4960). For western blotting, antibody α-tubulin (clone YL1/2, Bio-Rad, #MCA77G) was used. PCR amplicon purification was performed using the PureLink PCR purification kit (Thermo Fisher Scientific, #K310001). For mass spectrometry sample preparation, cell pellet homogenization was performed using Precellys Lysing Kit, Tissue homogenizing CK mix (Bertin Corp, #P000918-LYSK0-A). QUANTI-Blue reagent was used for interferon assays (InvivoGen, rep-qbs).

### Biological resources

A549 (human adenocarcinoma), Madin–Darby canine kidney (MDCK), and human embryonic kidney (HEK) 293T or 293 cells were cultured in Dulbecco's modified essential medium (DMEM) (Sigma-Aldrich) with 10% fetal calf serum (FCS) (Thermo Fisher) and 1% penicillin/streptomycin (Thermo Fisher) (growth medium) and were passaged twice weekly. A549 ZAP−/− cells were a gift from Professor Sam Wilson (Shaw et al. 2021). HEK 293 TRIM25−/− cells were a gift from Professor Gracjan Michlewski (Choudhury et al. 2022). A549 KHNYN−/− cells were a gift from Dr. Chad Swanson (Ficarelli et al. 2019). The knockout status of these cell lines was previously verified by our laboratory (Sharp et al. 2023). HEK-Blue IFNα/β cells were commercially acquired (InvivoGen , #hkb-ifnabv2).

### Plasmids and plasmid mutagenesis

IAV reverse genetics plasmids for the A/Puerto Rico/8/1934 (H1N1) (PR8) virus were derived from the UK National Institute of Biological Standards and Control strain (de Wit et al. 2004). The CpGH virus, with CpGs added into segment 5 of the PR8 strain, has been previously described (Gaunt et al. 2016). To generate a panel of reversion mutants with fragments of the CpGH region reverted to WT sequence, PCR and Gibson assembly were used. For PCR reactions, fragments of either the WT PR8 segment 5 plasmid, or seg5-CpGH plasmid, were amplified (as was the plasmid backbone, which is the same for both constructs; primers are listed in Table 2). The fragments generated are summarized in Figure 1A and Table 1. Generated fragments and backbone were added in 1:1 ratios directly to the Gibson assembly reaction mix equivalent to half the total reaction volume, and reactions were carried out in accordance with the manufacturer's instructions to generate new plasmids, confirmed by agarose gel electrophoresis. These were heat-shocked into DH5α chemically competent *Escherichia coli*, and grown on agar plates containing 50 µg/mL ampicillin selection. Picked colonies were sequenced using colony PCR, and plasmids containing the correct insert sequences were amplified using QIAGEN midi-prep kit, then checked again by Sanger sequencing.

**TABLE 2.** Site-directed mutagenesis primers targeting nucleotide positions 312 and 315 in PR8 segment 5

Target strain	Segment 5 mutation	Sequence
PR8 WT	T312A_Fw	GTCCTCCAGTTTTCTTTGGATCTTTCCCCGCAC
	T312A_Rev	GTGCGGGGAAAGATCCAAAGAAAACTGGAGGAC
	G315A_Fw	ATAGGTCCTCCAGTTTTTTTAGGATCTTTCCCCGC
	G315A_Rev	GCGGGGAAAGATCCTAAAAAAACTGGAGGACCTAT
	T312A G315A_Fw	TATAGGTCCTCCAGTTTTTTTTGGATCTTTCCCCGCACTG
	T312A G315A_Rev	CAGTGCGGGGAAAGATCCAAAAAAAACTGGAGGACCTATA
PR8 segment 5 CpGH	A312T_Fw	GGTCCTCCAGTTTTTTTAGGATCTTTACCCGCGCT
	A312T_Rev	AGCGCGGGTAAAGATCCTAAAAAAACTGGAGGACC
	A315G_Fw	ATGGGTCCTCCAGTTTTCTTTGGATCTTTACCCGC
	A315G_Rev	GCGGGTAAAGATCCAAAGAAAACTGGAGGACCCAT
	A312T A315G_Fw	AATGGGTCCTCCAGTTTTCTTAGGATCTTTACCCGCGCTC
	A312T A315G_Rev	GAGCGCGGGTAAAGATCCTAAGAAAACTGGAGGACCCATT

Single nucleotide changes in segment 5, corresponding to nucleotide positions 312 and 315, were made using site-directed mutagenesis. Primers are tabulated (Table 2).

Full segment sequences are summarized (Table 1).

For viral genome packaging assays, a positive control mutant, “4c6c,” was generated by synonymous mutagenesis of codons 546–548 of the PR8 HA gene and codons 451–453 of PR8 NA gene as previously described (Wise et al. 2019).

### Virus rescues

Virus rescues were performed as previously described (Hutchinson et al. 2008; Wise et al. 2009; Gaunt et al. 2016). Growth medium on HEK293T cells at 90% confluency in 6-well plates was replaced with Opti-MEM reduced serum medium (Thermo Fisher), and cells in each well were transfected with 250 ng each of eight pDUAL reverse genetics plasmids, one per segment of viral genome, in combination with 4 µL lipofectamine 2000 (Thermo Fisher). Mocks were transfected with seven plasmids, with segment 5 plasmid omitted. The next day, Opti-MEM was replaced with viral growth medium composed of DMEM containing 1 µg/mL tosyl phenylalanyl chloromethyl ketone (TPCK)–treated trypsin and 0.14% bovine serum albumin (w/v) (Sigma). After 48 h, supernatants containing viruses were collected and inoculated into the allantoic fluid of embryonated hens’ eggs at 10 days postfertilization (100 µL/egg). To generate the “4c6c” packaging mutant, virus stocks were instead propagated in MDCK cells rather than eggs to minimize the risk of reversion (Wise et al. 2019).

### Virus titrations

The amount of infectious virus in virus stocks and infection supernatants was quantified using plaque assays. Confluent MDCK cells in 12-well plates were inoculated with 300 µL volumes of 10-fold serial dilutions. After ∼1 h, cells were overlaid with viral growth medium diluted 1:1 in 2.4% cellulose (Sigma). Plaque assays were incubated for 48 hours, then 1 mL/well of 10% neutral buffered formalin was added to fix the cells for at least 20 min. Overlay was then discarded, and cells were stained using toluidine blue dye (0.1%) (Sigma).

### Virus infections

For viral growth assays and serial passage experiments, cells were infected at low MOI (0.01) and infected for 48 h in viral growth medium. For interferon induction, replication kinetics, and mass spectrometry experiments, high MOI (3) infections were performed for 8 h in serum-free medium. For all infections, virus was incubated on cells for ∼1 h, after which time (considered to be 1 h postinfection) cells were washed and the relevant medium was added.

### Virus purification and urea-PAGE gel electrophoresis

10^10^ plaque-forming unit (PFU) of egg-derived virus stocks were semipurified by pelleting through a 25% sucrose cushion (100 mM NaCl, 10 mM Tris-HCl pH 7.0, 1 mM EDTA) using centrifugation in a Beckman Coulter MAX-E ultracentrifuge at 280,000*g* for 90 min at 4°C with a SW 32 Ti rotor. Pelleted virions were resuspended into 350 µL RLT buffer, and RNAs were extracted. RNAs were loaded onto homemade 5% urea polyacrylamide gels in 1× Tris-borate-ETA (TBE) buffer (89 nM Tris-borate, 2 mM EDTA pH 8.3) and separated by electrophoresis at 120 V for 6 h. RNA was visualized using Bio-Rad Silver Stain Plus Kit and a Samsung Xpress C480FW scanner.

### In vitro transcription assay

To generate DNA input under a T7 promoter suitable for use in cell-free transcription assays, full-length segment 5 was amplified by PCR using primers with a T7 promoter sequence added to the 5′ end of the forward primer. Q5 DNA polymerase kit was used to make amplicons, with 25 ng plasmid and an initial incubation of 95°C for 5 min, followed by 30 cycles of 95°C for 30 sec, 50°C for 30 sec, and 72°C for 2 min, then a 72°C for 5 min final extension. PCR products were run on an agarose gel, then bands of the expected size were excised and gel purified. Forty nanograms of amplicon was used as template in MEGAscript T7 transcription assays with half-reaction volumes, for 10, 20, 45, or 90 min. Reactions were terminated, and RNA was quantified using Qubit RNA HS Assay.

### In vitro translation assay

RNA transcripts generated as above were used as input templates for cell-free translation assays. Transcend Biotin-Lysyl-tRNA (Promega) was incorporated in reactions, so that reaction products could be identified using western blotting as below, except that membranes were blocked using 5% BSA/TBS for 60 min, then incubated with IRDye 800CW Streptavidin (LICOR) for a further 60 min, and then imaged using LICOR Odyssey Fc imaging system.

### Western blotting

Cell lysates from each well of a 24-well plate were harvested in 100 µL Laemmli buffer (2×) and boiled for 10 min. Samples were cooled, and 5 µL/well was loaded into 10% polyacrylamide precast gels (Bio-Rad) and SDS-PAGE was performed. Resolved proteins were wet-transferred onto nitrocellulose membranes (Thermo Fisher Scientific) at 100 V for 90 min. Membranes were blocked for 30 min with 5% skimmed milk powder diluted in PBS (w/v) and 0.1% Tween-20. Membranes were probed with in-house NP, PB2 (Carrasco et al. 2004) (1:1000), and β-tubulin (1:5000) antibodies at 4°C overnight, washed three times, and then incubated with 1:5000 Alexa Fluor 680 or 800 species-specific antibodies (Thermo Fisher Scientific) for 90 min. After three washes, membranes were visualized on a LICOR Odyssey Fc imaging system.

### Northern blotting

Northern blotting was performed as previously described (Sharp et al. 2023). Briefly, A549 cells were infected at MOI 3 for 8 h. Cells were collected, RNA was extracted, then separated by urea-PAGE electrophoresis. RNA was transferred onto nylon membrane overnight, then membranes were baked at 68°C for 10 min followed by UV cross-linking. Positive control segment 1 and segment 5 transcripts were generated using in vitro transcription assays. Membranes were hybridized overnight with biotinylated probes, then washed, blocked, washed, and incubated with HRP-conjugated streptavidin for 30 min. Membranes were washed and then visualized by exposure to chemiluminescent film. To examine RNA degradation, A549 cells were infected at MOI 10 for 24 h in the presence of 25 µg/mL cycloheximide to prevent protein synthesis and thus the formation of new vRNPs. Thus, any new positive sense transcripts synthesized were produced by input vRNPs and would be subject to turnover mediated by cellular proteins including ZAP. RNAs were harvested from these cells, and viral transcripts were visualized as described.

### Serial passage

The virus panel was serially passaged in A549 cells at MOI 0.01. Four biological repeats were passaged 10 times, with two repeats derived from egg-based virus rescues and two from MDCK-based virus rescues. Viruses were sequenced at passage 10 as previously described (Sharp et al. 2023) with the exception that Seg5 CpGH specific segment 5 primers (amplicons A, B, C, and D, Table 3) were included in place of the WT PR8 specific segment 5 primers in the pool. Earlier passages were sequenced using set of primers for segment 5 only in six overlapping amplicons. These PCRs were performed with the specified primer sets (a, c, and e or b, d, and f). Cycling conditions were 98°C for 30 sec and then 30 cycles of 98°C for 20 sec, 55°C for 20 sec, then 72°C for 30 sec with a final extension of 72°C for 2 min. Amplicons were purified and subjected to a second PCR round to add partial Illumina sequences and barcodes using the primers in Table 3. Cycling conditions were 98°C for 30 sec, then 10 cycles of 98°C for 20 sec, 55°C for 20 sec, then 72°C for 30 sec with a final extension of 72°C for 2 min. Amplicons were gel purified and sent for sequencing using the Amplicon-EZ service (GENEWIZ). Sequencing data handling and analyses were performed using the Galaxy platform (Afgan et al. 2018). Primer sequences were trimmed from reads using *cutadapt*, and output sequences were joined using *fastq-join*. Sequences were aligned to the CpGH recoded segment 5 using *bowtie2* (Langmead et al. 2009). Variants and coverage levels in the resultant BAM data sets were analyzed using *iVar* variants with tabular outputs (Grubaugh et al. 2019).

**TABLE 3.** Primers used to generate PCR amplicons

Primer name	Sequence	Target fragments/function
pDual_backbone_Fw	GAGTGATTATCTACCCTGCTTTTGCT	Complete segment
pDual_backbone_Rev	GAGTACGACAATTAAAGAAAAATACCCTTGTTTCTACT	Complete segment
T7 Seg5 +sense_Fw	TAATACGACTCACTATAGGGAGCAAAAGCAGGGTAGATAATCA	Complete segment
T7 Seg5 +sense_Rev	AGTAGAAACAAGGGTATTTTTCTTTAATTGT	Complete segment
T7 Seg5 −sense_Fw	AGCAAAAGCAGGGTAGATAATCA	Complete segment
T7 Seg5 −sense_Rev	TAATACGACTCACTATAGGGAGTAGAAACAAGGGTATTTTTCTTTAATTGT	Complete segment
Seg5_302-329_Fw	GGAAAGATCCTAAGAAAACTGGAGGACC	D
Seg5_598-624_Fw	GGAGTTGGAACAATGGTGATGGAATTG	A, B
Seg5_755-780_Fw	CAATGATGGATCAAGTGAGAGAGAGC	D, E
Seg5_959-986_Fw	GACTGCTTCAAAACAGCCAAGTGTACAG	B, C
Seg5_1256-1283_Fw	GCCAAATCAGCATACAACCTACGTTCTC	E
Seg5_1565-1528_Rev	AGTAGAAACAAGGGTATTTTTCTTTAATTGTCGTACTC	A, B, C, D, E
Seg5_1283-1256_Rev	GAGAACGTAGGTTGTATGCTGATTTGGC	E
Seg5_986-959_Rev	CTGTACACTTGGCTGTTTTGAAGCAGTC	B, C
Seg5_780-755_Rev	GCTCTCTCTCACTTGATCCATCATTG	D, E
Seg5_624-598_Rev	CAATTCCATCACCATTGTTCCAACTCC	A, B
Seg5_329-302_Rev	GGTCCTCCAGTTTTCTTAGGATCTTTCC	D
Seg5 CpGH amplicon A	CAAAAGCAGGGTAGATAATC	Seg5 serial passage
Seg5 CpGH amplicon A	TCGTCACCATTGTTCGCTTG	Seg5 serial passage
Seg5 CpGH amplicon B	GCGCGAACTAATACTTTACG	Seg5 serial passage
Seg5 CpGH amplicon B	TTACGGCTCTCTCTAACTTG	Seg5 serial passage
Seg5 CpGH amplicon C	AAAATTTCAAACCGCGGCGC	Seg5 serial passage
Seg5 CpGH amplicon C	TTTGAAGCTATTTGAACGCC	Seg5 serial passage
Seg5 CpGH amplicon D	CATAAAAGGAACGAAAGTCC	Seg5 serial passage
Seg5 CpGH amplicon D	GTACTCCTCTGCATTGTCTC	Seg5 serial passage
IlluminaTag1F	ACACTCTTTCCCTACACGACGCTCTTCCGATCTAACACATCGTAAAACGACGGCCAGT	Deep sequencing
IlluminaTag3F	ACACTCTTTCCCTACACGACGCTCTTCCGATCTCCGTATATGTAAAACGACGGCCAGT	Deep sequencing
IlluminaTag5F	ACACTCTTTCCCTACACGACGCTCTTCCGATCTGAGATAACGTAAAACGACGGCCAGT	Deep sequencing
IlluminaTag8F	ACACTCTTTCCCTACACGACGCTCTTCCGATCTTGCTCCGAGTAAAACGACGGCCAGT	Deep sequencing
IlluminaTagR	GACTGGAGTTCAGACGTGTGCTCTTCCGATCTCAGGAAACAGCTATGAC	Deep sequencing
Seg5 PR8_F	GTAAAACGACGGCCAGTTCTGCTTTTGACGAAAGGAG	Slippage sequencing
Seg5 PR8_R	CAGGAAACAGCTATGACAACCTTGCATCAGAGAGCAC	Slippage sequencing
Seg5 CDLR_F	GTAAAACGACGGCCAGTTCTGCTTTTGACGAAAGAAG	Slippage sequencing
Seg5 CDLR_R	CAGGAAACAGCTATGACAGCCTTGCATCAGAGAGCAC	Slippage sequencing
Reporter GFP_F	AGGGTGACAAAAACATAGGATCC	Slippage sequencing
Reporter GFP_R	CAGGGCACGGGCAGCTTGC	Slippage sequencing

### Slippage PCR

RNA was extracted from transfected or infected cells or infected culture supernatants. Extracted RNAs were reverse transcribed using SuperScript III reagents (Invitrogen) with the IAV gRNA specific Uni12 primer (AGCAAAAGCAGG), cRNA/mRNA specific Uni13 primer (AGTAGAAACAAGG) (Hoffmann et al. 2001), or Reporter GFP_R primer (CAGGGCACGGGCAGCTTGC) according to the manufacturer's instructions. PCRs were performed using specific reaction primer pairs specific to the appropriate parental segment (Table 3) and Q5 High-Fidelity Polymerase (NEB) according to the manufacturer's instructions. Cycling conditions were 98°C for 30 sec and then 30 cycles of 98°C for 20 sec, 55°C for 20 sec, and 72°C for 30 sec with a final extension of 72°C for 2 min. Amplicons were sent for sequencing using the Amplicon-EZ service (GENEWIZ) or Sanger sequencing with the reverse primer. Deep amplicon sequencing data handling and analyses were performed using the Galaxy platform (Afgan et al. 2018). Primer sequences were trimmed from reads using *cutadapt*, and output sequences were joined using *fastq-join*. Sequences were aligned against reference genome data sets against the canonical nucleotide 312–319 region using *bowtie2* (Langmead et al. 2009). Coverage levels in the resultant BAM data sets were analyzed using *Samtools depth*. Sanger sequencing traces were visualized using Chromas v2.5.1.

### Mass spectrometry

To test whether the poly(A) tract introduced into the CpGH virus resulted in the production of a frameshifted peptide, ∼1 × 10^6^ A549 cells were infected at MOI 10 for 8 h in the presence or absence of 10 µM MG132 protease inhibitor. The cells were trypsinized, spun down, and stored immediately at −80°C until further processing. The cell pellets were resuspended in an extraction buffer containing 5% SDS, 50 mM triethyl ammonium bicarbonate (TEAB), pH 8.5 at sample to buffer ratio of 1:10 (w/v) and homogenized using Precellys homogenizer at 5000*g* for 20 sec in a ceramic beads vial. The extracts were centrifuged for 10 min at 16,000*g*, and supernatant was sonicated for 10 cycles with 30 sec on and 30 sec off per cycle on a Bioruptor Pico Sonicator (Diagenode). After sonication, samples were centrifuged (16,000*g* for 10 min), supernatant was collected, and a BCA assay was performed.

### S-Trap proteolytic digestion and LC-MS

The samples were digested on the S-Trap micro column (ProtiFi) following the manufacturer's protocol with minor modifications. Briefly, 20 μg protein in extraction buffer was reduced and alkylated using 10 mM dithiothreitol and 40 mM iodoacetamide, respectively, at 45°C for 15 min. Alkylation was stopped by adding phosphoric acid to a final concentration of 2.5%, followed by sixfold volume of binding buffer (90% v/v, methanol in 100 mM TEAB) to the protein solution. After gentle vertexing, the colloidal protein solution was loaded into an S-Trap micro column. The solution was removed by spinning the column at 4000*g* for 1 min. The column was washed with 150 μL binding buffer three times. Due to the 8A stretch introducing multiple lysines, this meant that the standard approach of tryptic cleavage would not allow us to distinguish between +1 and −2 or +2 and −1 frameshift events, and so gluC protease that cleaves downstream from glutamic acid was used instead. Therefore, 20 μL of digestion solution (1 μg GluC in 50 mM TEAB) was added to the column and incubated at 47°C for 2 h. Digested peptides were eluted using 40 μL of three buffers consecutively: (i) 50 mM TEAB, (ii) 0.2% (v/v) formic acid in H_2_O, and (iii) 50% (v/v) acetonitrile. Eluted peptides were pooled and cleaned up using C_18_ stagetips and dried under vacuum. Purified peptides were separated over a 70 min gradient on an Aurora-25 cm column (IonOpticks) using an UltiMate RSLCnano LC System (Thermo Fisher Scientific) coupled to a timsTOF FleX mass spectrometer via a CaptiveSpray ionization source. The gradient was delivered at a flow rate of 200 nL/min. The column temperature was set at 50°C. For DDA-PASEF acquisition, the full scans were recorded from 100 to 1700 m/z spanning from 1.45 to 0.65 Vs/cm^2^ in the mobility (1/*K*_0_) dimension. Up to 10 PASEF MS/MS frames were performed on ion-mobility separated precursors, excluding singly charged ions, which are fully segregated in the mobility dimension, with a threshold and target intensity of 1750 and 14,500 counts, respectively. Raw mass spectral data were processed using PEAKS Studio X-Pro Software (Bioinformatics Solutions Inc.). Search was performed against UniProt human sequence database containing 20,586 entries appended with PR8 protein sequences at an MS1 precursor tolerance of 20 ppm and MS2 tolerance of 0.06 Da. Full Glu-C digestion allowing one missed cleavage, fixed modification of cystine [+57.02], oxidation of methionine, deamination of asparagine and glutamine were also specified as variable modifications for database search.

### HEK Blue assay

Type I IFN production was measured using HEK Blue reporter assays. Subconfluent A549 cells in 24-well plates were infected at MOI 10 for 10 h. Supernatants were then harvested and UV treated to inactivate virus by exposing to 120 mJ/cm^2^ in a UVP CL-1000 UV cross-linker for 10 min. Twenty microliters of UV-treated sample was inoculated onto 4 × 10^4^ cells/well of HEK-Blue IFNα/β cells in 96-well plates, or titrated human recombinant IFN-β as control (5, 50, or 500 pg/µL). Cells were incubated at 37°C for 24 h before supernatants were collected and mixed with QUANTI-Blue reagent. Color changes, visible after 15–30 min and reflective of the amount of IFN present, were measured by reading absorbance at 620 nm.

### GFP reporter assay

Two plasmid constructs were designed to encode the EGFP ORF downstream from an 8-adenosine or 8-uracil tract, flanked by PR8 NS1 segment UTRs that are recognized by the viral polymerase and under a pol.I promoter. These constructs were used in a minigenome assay to determine whether polymerase slippage could be evidenced through reporter gene detection. Production of the negative sense reporter GFP transcript with IAV UTRs allows transcription of the gene into the positive orientation by IAV polymerase. However, due to the incorporation of the 8A/8U tract upstream of the GFP ORF, the ORF is out of frame. Polymerase slippage on the 8A/8U such that a single nucleotide insertion arises will give a transcript with 9A/9U upstream of the GFP, thereby putting the ORF in frame and yielding GFP signal. Thus, GFP production can only occur if polymerase slippage occurs. Reconstitution of the viral polymerase was achieved by cotransfection of the reporter plasmid into HEK293T cells with an additional plasmid encoding the PB2, PB1, PA, and NP proteins of PR8, (segments 1, 2, 3, and 5 respectively). For experiments comparing the polymerase activity between different strains, reporter constructs were cotransfected with four individual plasmids separately encoding PB2, PB1, PA, and NP proteins of PR8, A/California/04/2009 (H1N1) (Cal04), A/mallard/Netherlands/10-Cam/1999 (H1N1) (Mallard), A/swine/England/87842/1990 (H3N2) (Swine), and A/Udorn/307/1972 (H3N2) (“Udorn). GFP signal was read at 0, 24, 48, and 72 h post-transfection on a Cytation 3 plate reader.

### Data availability

Sequencing data generated during this work have been uploaded to the NIH BioSample Repository and are available under accession numbers SAMN49853699–SAMN49853709 (slippage amplicon sequencing) and SAMN49886313–SAMN49886326 (passaged virus amplicon sequencing).

### Statistical analyses

All experiments were performed in at least biological triplicate unless otherwise stated, and statistical analyses were only performed on data representative of at least three biological repeats. One-way analysis of variance (ANOVA) tests were performed to assess differences across groups under the same experimental conditions using GraphPad Prism 9. Statistical significance is indicated throughout as (*) *P* < 0.05, (**) *P* < 0.01, (***) *P* < 0.001, (****) *P* < 0.0001. Error bars indicate standard error.

## DATA DEPOSITION

The data underlying this article will be shared on reasonable request to the corresponding author.

## Figures and Tables

**Colin P. Sharp:**
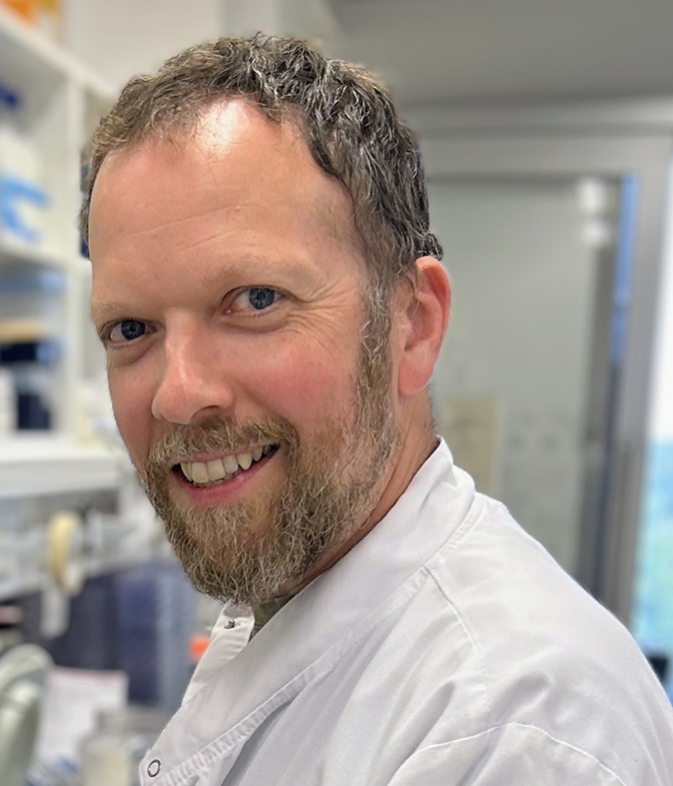


## MEET THE FIRST AUTHOR

***Meet the First Author(s)* is an editorial feature within *RNA*, in which the first author(s) of research-based papers in each issue have the opportunity to introduce themselves and their work to readers of *RNA* and the RNA research community. Colin P. Sharp is the first author of this paper, “Understanding off-target growth defects introduced to influenza A virus by synonymous recoding.” Colin is a postdoctoral researcher (core scientist) working in the lab of Dr. Elly Gaunt at the Roslin Institute (University of Edinburgh). He works on many aspects of influenza A virus biology and is currently focused on understanding the compositional biases of the viral genome.**

**What are the major results described in your paper, and how do they impact this branch of the field?**

We initially discovered that a synonymously recoded viral mutant was not attenuated for the reasons intended by its design, namely recognition of the additional CpG dinucleotides by the cellular sensor ZAP. In identifying the recoded region that was responsible for the attenuation, we were led to the discovery that the influenza polymerase can “slip” on poly(A) and poly(U) tracts within a viral coding region causing misincorporation of nucleotides and a subsequent production of aberrant proteins. This work will have a direct impact in helping to characterize the constraints upon recoding-mediated viral attenuation for vaccine development. There are also a number of broader impacts to the field including a deeper understanding of influenza polymerase biology and viral evolution.

**What led you to study RNA or this aspect of RNA science?**

As with all viruses with an RNA genome, RNA is central to every process in the influenza replication cycle. With my primary focus being the investigation of compositional biases in the viral genome, a better understanding of how the viral RNA interacts with host and viral proteins is key to being able to apply this knowledge for improved vaccine design as well as exploring fundamental questions about viral biology.

**During the course of these experiments, were there any surprising results or particular difficulties that altered your thinking and subsequent focus?**

When we had initially identified the poly(A)/poly(U) tract as the region responsible for the attenuation of the recoded virus, there was a suspicion that the effects we were seeing were due to ribosomal slippage. However, when I sequenced the mRNA, I could see clear evidence of slippage and misincorporation of additional nucleotides by the viral polymerase but not host polymerase. This was a bit of a surprise but, as I am personally a big fan of sequencing and playing around with sequencing data, this was also a very welcome finding as it gave me the opportunity to apply further sequencing techniques to the subsequent work.

**What are some of the landmark moments that provoked your interest in science or your development as a scientist?**

My interest in science was first piqued in a high school microbiology class. I was fascinated not just by the idea of getting interesting results but by the techniques and reagents used to get them. This has continued throughout my career, and I'm still just as excited about working up a new method or using a different piece of equipment as I am about the results I get. Another key moment was when I started working on viruses during my PhD studies with Dr. Bob Dalziel. I have always been astounded by their ability to do so much with so little. This was further reinforced when I discovered my first novel anellovirus. Not only was it incredibly exciting seeing the genome of a new virus for the first time, but the fact that it was only ∼2 kb was really astounding.
